# Impact of socioeconomic inequalities on geographic disparities in cancer incidence: comparison of methods for spatial disease mapping

**DOI:** 10.1186/s12874-016-0228-x

**Published:** 2016-10-12

**Authors:** Juste Aristide Goungounga, Jean Gaudart, Marc Colonna, Roch Giorgi

**Affiliations:** 1 Aix Marseille University, INSERM, IRD, SESSTIM, Sciences Economiques & Sociales de la Santé & Traitement de l’Information Médicale, Marseille, France; 2 APHM, Hôpital de la Timone, Service Biostatistique et Technologies de l’Information et de la Communication, Marseille, France; 3 Registre des cancers de l’Isère, CHU de Grenoble, F-38000 Grenoble, France

**Keywords:** Spatial analysis, Cluster detection, Cancer, Oblique decision tree

## Abstract

**Background:**

The reliability of spatial statistics is often put into question because real spatial variations may not be found, especially in heterogeneous areas. Our objective was to compare empirically different cluster detection methods. We assessed their ability to find spatial clusters of cancer cases and evaluated the impact of the socioeconomic status (e.g., the Townsend index) on cancer incidence.

**Methods:**

Moran’s I, the empirical Bayes index (EBI), and Potthoff-Whittinghill test were used to investigate the general clustering. The local cluster detection methods were: i) the spatial oblique decision tree (SpODT); ii) the spatial scan statistic of Kulldorff (SaTScan); and, iii) the hierarchical Bayesian spatial modeling (HBSM) in a univariate and multivariate setting. These methods were used with and without introducing the Townsend index of socioeconomic deprivation known to be related to the distribution of cancer incidence. Incidence data stemmed from the Cancer Registry of Isère and were limited to prostate, lung, colon-rectum, and bladder cancers diagnosed between 1999 and 2007 in men only.

**Results:**

The study found a spatial heterogeneity (*p* < 0.01) and an autocorrelation for prostate (EBI = 0.02; *p* = 0.001), lung (EBI = 0.01; *p* = 0.019) and bladder (EBI = 0.007; *p* = 0.05) cancers. After introduction of the Townsend index, SaTScan failed in finding cancers clusters. This introduction changed the results obtained with the other methods. SpODT identified five spatial classes (*p* < 0.05): four in the Western and one in the Northern parts of the study area (standardized incidence ratios: 1.68, 1.39, 1.14, 1.12, and 1.16, respectively). In the univariate setting, the Bayesian smoothing method found the same clusters as the two other methods (RR >1.2). The multivariate HBSM found a spatial correlation between lung and bladder cancers (*r* = 0.6).

**Conclusions:**

In spatial analysis of cancer incidence, SpODT and HBSM may be used not only for cluster detection but also for searching for confounding or etiological factors in small areas. Moreover, the multivariate HBSM offers a flexible and meaningful modeling of spatial variations; it shows plausible previously unknown associations between various cancers.

**Electronic supplementary material:**

The online version of this article (doi:10.1186/s12874-016-0228-x) contains supplementary material, which is available to authorized users.

## Background

Statistical methods that assess the impact of a spatial structure on the occurrence of a particular health event have been developed in many areas over the recent decades [1]. These methods allow detecting clusters of disease cases and mapping observations or estimations [2]. They combine techniques from geography, epidemiology, and public health to better understand health needs and allocate resources.

Currently, various epidemiological information systems are used to collect and analyze health-related data and guide political decisions. In France, cancer cases are collected by the Institut National de Veille Sanitaire and the Institut National du Cancer in collaboration with Francim network of cancer registries [2]. Mapping cancer cases may have a significant impact on the perception of excess rates in particular regions, but the heterogeneity in population density between administrative areas may affect the interpretation of mapping results, especially in case of small areas [3]. To display this heterogeneity, maps may be produced by operating transformations that better reflect the spatial distribution of the disease [1]. Still, whatever the parametric or non-parametric method used to account for uncertainty in the spatial distribution of the disease, the choice of the measure to be shown remains a key issue [4].

In a given geographical area, some health indicators may reveal an excess number of cases. This excess is often estimated by the standardized incidence ratio (SIR) [5]. Generally, the SIR estimates the risk of disease in a given spatial unit and depends on the existence of either a spatial autocorrelation (i.e., lack of independence between observations) or a spatial heterogeneity. Comparisons of SIRs between neighboring spatial units may suggest grouping sets of spatial units into classes or clusters.

Two types of methods may be used to detect disease clusters; i.e., aggregates of cases [6]. Local methods are able to detect and locate clusters with or without a predetermined source point. In addition to spatial location, some local methods allow for confounding factors that may affect the spatial distribution of the disease [7]. Global methods look for the presence –but not the location– of a clustering pattern [8, 9]. For example, Moran’s autocorrelation coefficient is a global method that measures the spatial autocorrelation weighted by a function of the distance between two close points defined by their centroids (“average X, average Y”) [10].

Several local detection methods allow identifying clusters with particular shapes within the study area: i) the spatial scan statistic of Kulldorff (SaTScan) [11] performs one or several circular or elliptic scans; (ii) regression trees such as Spatial Oblique Decision Tree (SpODT) perform oblique cuts; and, iii) hierarchical Bayesian spatial modeling (HBSM) [4] produces a real smoothing of the SIR. Many Bayesian applications have been already used in infectious diseases and cancer; they were able to distinguish random fluctuations from true changes in the incidence of the disease.

The first applications of SpODT were made in the field of infectious diseases; they contributed to the detection of spatial classes, for example, different risks of malaria in a Malian village [12] and a spatial pattern of Buruli disease in Cameroon [13]. In comparison with SaTScan and classification regression trees (CART), SpODT provided complementary information, and, in some cases, was more accurate [12, 14]. However, SpODT has never been applied to detection of clusters of cancer cases.

Concerning the geographic distribution of cancer cases, spatial clustering seems to exist in lung, prostate, bladder, and colon-rectum cancers but this clustering depends mainly on the available data [15–18]. Furthermore, the clustering of cancer in a given area may depend on factors such as the socioeconomic status [19] and on unknown risk factor common to other diseases [20].

The main objective of the present study was to compare empirically different cluster detection methods by assessing their abilities to find spatial clusters of cancer cases. Secondarily, the study aimed also to evaluate the impact of the Townsend index of socioeconomic status on cancer incidence.

Using global detection methods with data on four cancers, we sought first, for the presence of particular spatial patterns. Then, we compared the results with those obtained with local methods (SaTScan, SpODT, and HBSM) with and without taking into account a confounding factor; the Townsend index. Thereafter, we compared the abilities of the three approaches to estimate random changes in the incidence of each cancer. Finally, using a multivariate HBSM, we examined whether factors common to the four cancers could increase the reliability of the results.

## Methods

### Population and area

Incident cancer cases diagnosed between 1998 and 2007 were all extracted from the Cancer Registry of Isère (for more details on this registry, see Ref. [5]). The study considered only prostate, lung, colon-rectum, and bladder cancers in men. The confounding factor was the Townsend index of deprivation, an indicator of socioeconomic disadvantage. This index is a synthesis of the following variables: proportion of unemployed people in the workforce, proportion of households without car, proportion of households renting, and the proportion of overcrowded households. This index increases with the increase in the level of deprivation [14, 21]. The spatial unit was the Commune; i.e., the smallest administrative unit in France (mean number of inhabitants: 1700 over France, 2300 over Département Isère in 2006) [22].

### Statistical analysis strategy

First, we searched for the presence of spatial heterogeneity using the Potthoff-Whittinghill method [23–25]. Briefly, if there is no clustering, the observed number of cases in a geographical area should follow a Poisson distribution (mean = variance = expected number of cases in the area). The Potthoff-Whittinghill test checks whether the ratio of the variance to the expected number of cases is >1, in which case the data are said over-dispersed relative to the Poisson distribution (See further details below).

The “naive” global spatial autocorrelation was checked using Moran’s I statistic and the “true” global spatial autocorrelation was confirmed using the Empirical Bayes Index. Another way to measure the spatial effect or the spatial correlation in each cancer type was to compare the goodness of fit between spatial and non-spatial regression models. For this, we used two non-spatial models, the Poisson regression and the constant risk model. Thereafter, we used spatial heterogeneity, autocorrelation, and Besag-York-Mollié (BYM) models in a Bayesian approach [26]. We also used SaTScan and SpODT to explore their abilities to detect spatial clusters in different scenarios (homogeneity, heterogeneity, spatial autocorrelation and the latter two) also detected by global detection methods. The Townsend socioeconomic index was then introduced into the modeling of all local detection methods in the univariate setting to assess its impact on the spatial distribution of each cancer type. Finally, we used a multivariate Bayesian modeling to assess the impact of underlying correlations between the four cancers on their incidences.

### Statistical analysis

#### Poisson non-spatial regression model

Poisson regression model assumes that *O*_*i*_|*θ*_*i*_ ∼ *Poisson*(*E*_*i*_ *θ*_*i*_); *O*_*i*_ being the number of cases, *θ*_*i*_ the relative risk, and *E*_*i*_ the expected number of cases in a given area *i*. The SIR is the maximum likelihood estimator of *θ*_*i*_ and is given by $$ SI{R}_i=\raisebox{1ex}{${Y}_i$}\!\left/ \!\raisebox{-1ex}{${E}_i$}\right. $$. The variance of this model, $$ \raisebox{1ex}{$SI{R}_i$}\!\left/ \!\raisebox{-1ex}{${E}_i$}\right. $$ is large when *E*_*i*_ is small, which reflects an over-dispersion of cases. The Poisson model assumes also that $$ \log \left({\theta}_i\right)= log\left({E}_i\right)+\beta {\mathcal{X}}_i;\;\mathcal{X} $$ being a covariate and *β* its effect.

Incidence data regarding the four cancer types were first fitted with Poisson regression and using the additional information on the socioeconomic status.

#### Global detection method

The global spatial autocorrelation analysis was carried out by type of cancer using Moran’s I statistic and the Empirical Bayes Index (EBI), an adapted Moran’s I proposed to take into account a heterogeneity in population distribution.

$EBI=\frac{N}{{\displaystyle \sum }{w}_{ij}}\frac{{\displaystyle \sum }{w}_{ij}{z}_i{z}_j}{{\displaystyle \sum }{\left({z}_i-\overline{z}\right)}^2}$ where N is the number of Communes, ${z}_i=\frac{p_i-b}{\sqrt{v_i}}$. and ${p}_i=\frac{O_i}{x_i}$, *O*_*i*_ being the number of cases and *x*_*i*_ the population at risk in Commune ${C}_i,\kern0.5em {v}_i=a+\left(\frac{b}{x_i}\right),\;b=\frac{O}{x},\;a={s}^2-\frac{b}{\left(\frac{x}{N}\right)},\;{s}^2={\displaystyle \sum }{x}_i\frac{{\left({p}_i-b\right)}^2}{x}$.

Spatial heterogeneity was tested with Potthoff-Whittinghill method, the null hypothesis being that the numbe observed cases *O*_*i*_. in a Commune *C*_*i*_ is Poisson distributed and the mean being the number of expected cases *E*_*i*_. The test statistic may be written as follows: $PW={\displaystyle {\sum}_i^N\frac{O_i\left({O}_i-1\right)}{E_i}}$ which is asymptotically normally distributed, with mean $\frac{O_{+}\left({O}_{+}-1\right)}{E_{+}}$ and variance $2\left(N-1\right)\frac{O_{+}\left({O}_{+}-1\right)}{{\left({E}_{+}\right)}^2}$ and where N is the number of Communes *C*_*i*_, *O*_+_ = ∑_*i* = 1_^*N*^ *O*_*i*_, and *C*_+_ = ∑_*i* = 1_^*N*^ *C*_*i*_.

Moran’s I and Potthoffhittinghill statistics were computed using Monte-Carlo simulations with 999 replications [14] under the assumption of multinomial distribution of cancer cases. The EBI value was tested using 999 random permutations. Considering the Commune as the spatial unit, three clustering methods were investigated and compared: SpODT, SaTScan, and HBSM.

#### SpODT method

This is a non-parametric regression model (similar to the Classification and Regression Tree (CART) algorithm of Breiman [12]) that allows local detection of clusters. However, whereas CART provides perpendicular area cuts, SpODT provides oblique area cuts which are more suitable for spatial epidemiology [12, 27]. The functional form of the SpODT model is *z*_*i*_ = *f*(*x*_*i*_, *y*_*i*_) + *ε*_*i*_. In this formula, {*x*_*i*_, *y*_*i*_} correspond to the Commune centroids and *ε*_*i*_ ∈ ℝ represents the residuals. The functional form *f*(*x*_*i*_, *y*_*i*_) may be written:

$$ f\left({x}_i,{y}_i\right)={\displaystyle \sum_{j=1}^P}{\overline{z}}_j\ I\left\{{M}_i\left({x}_i,{y}_i\right)\in class\ j\right\} $$

where class *j* (*j* = 1, …, *P*) corresponds to one of the final *P* classes after splitting the area under study. For each point *M*_*i*_ in the *j* ^th^ class, ${\overline{z}}_j$ is the mean of the SIR values of all spatial units and ${z}_i={\overline{z}}_j\pm {\varepsilon}_i$ is the predicted risk.

SpODT algorithm makes recursively oblique cuts of the study area according to the overall interclass variance until reaching a final number *P* of areas as per the stopping criteria of the algorithm. Once the classification is obtained, a test is performed using a Monte-Carlo approach to compare the distribution with the estimated R2 [27]. For application to our data, we conducted a sensitivity analysis by changing the values of the parameters that serve as stopping criteria for the SpODT algorithm without using additional information on the socioeconomic status. After the univariate analysis, a multivariate analysis was performed and the SIRs of the detected patterns were those with *p*-value ≤0.05. Afterwards, we conducted further analyses, first varying the graft level setting to gather the adjacent final classes according to their similarity in terms of risk level, then adding the Townsend index to the model.

#### SaTScan

This method derives from the Geographical Analysis Machine (GAM) [28]. It aims at grouping neighboring spatial units into potential clusters through circular or elliptical windowing that scans the study area [11]. The observed number of cases is compared with the expected number inside and outside each window by computing, for each scan, a likelihood ratio statistic. Assuming that the observed data follow a Poisson model, the likelihood ratio is:

$$ \frac{L(Z)}{L_0}=\frac{{\left(\frac{n_z}{u(z)}\right)}^{n_z}{\left(\frac{N-{n}_z}{N-u(z)}\right)}^{N-{n}_z}}{{\left(\frac{N}{u(A)}\right)}^N} $$

In this formula, n_z_ and u(z) are the observed and expected numbers of cases in a circular frame Z, N and u(A) the observed and expected numbers of cases estimated under the null hypothesis of a homogeneous risk over the whole area, and *n*_z_>*u*(*z*) or equal to $$ \raisebox{1ex}{$1$}\!\left/ \!\raisebox{-1ex}{${\mathrm{L}}_{0\ }\ $}\right. $$. In the present study, we considered only circular windows because they detect smaller and more compact clusters than elliptical ones [29]. We conducted a sensitivity analysis with different maximum cluster sizes for the at-risk population; precisely, 1 to 50 % of the whole population. The analyses were performed with and without additional information on the socioeconomic status. The candidate clusters and their relative risks (RR) were those with a *p*-value ≤0.05 as obtained by Monte-Carlo simulation.

#### Univariate HBSM

This approach aims at modeling the spatial distribution and estimating the relative risk (*θ*_*i*_) at each spatial unit *i*. This approach allows taking into account spatial and non-spatial effects as structured information that can be graphically represented by four levels. The first level represents the local variability inside each spatial unit as provided by the observed data assumed having a Poisson distribution; this level gives the likelihood. The second level (or spatial process level) represents the variability between spatial units and depends on the presence of an autocorrelation $\left(\mathcal{U}\right)$ or a spatial heterogeneity $\left(\mathcal{V}\right)$. At this level, potential covariates $\left(\mathcal{X}\right)$ are assumed to be normally distributed. The third level (or priors) represents the variability of the spatial process components and is approached with a particular distribution (Beta, Gamma, Dirichlet, or Wishart) [30]. Here, a gamma distribution was chosen for precision (inverse of the variance). The parameters (called hyperparameters) of this distribution are fixed a priori according to previous recommendations [31]; they represent the fourth level. These hyperparameters were estimated using MCMC sampling and approximation methods.

Three a priori assumptions on the spatial process level were tested: i) autocorrelation with conditionally auto-regressive model (CAR model), analytically written $\log \left(\theta i\right) = \alpha +{\mathcal{U}}_i$; ii) heterogeneity with $\log \left(\theta i\right) = \alpha +{\mathcal{V}}_i$; iii) both, with $\log \left({\theta}_i\right) = \alpha +{\mathcal{U}}_i+{\mathcal{V}}_i$ according to the BYM model [26].

In the spatial process, the hypothesis of a homogeneous risk was tested with log(*θ*_*i*_) = *α*, which corresponds to the intercept [4, 5, 32]. Analyses were then performed using the additional information on the socioeconomic status $\left({\mathcal{X}}_i\right)$ with each previous model. In the BYM, this additional variable was written: $\log \left({\theta}_i\right)=\alpha +\beta *{\mathcal{X}}_i+{\mathcal{U}}_i+{\mathcal{V}}_i$.

To measure the influence of the socioeconomic status on the spatial distribution of cancer cases, we compared the models according to two criteria: i) the empirical variance of the autocorrelation components ${\mathcal{S}}_{\mathcal{U}}^2$; and, ii) heterogeneity components ${\mathcal{S}}_{\mathcal{V}}^2$ and their Deviance Information Criterion (DIC) [33]. A comparison was made between the DIC in each cancer type to identify the advantages of using a Bayesian approach. As in Colonna and Sauleau [4, 5, 32], we considered that a DIC difference of more than 10 points indicates the presence of an influence of the socioeconomic status on the differences in cancer incidence. The presence of either autocorrelation or heterogeneity was decided according to the model that had the highest empirical variance.

#### Multivariate HBSM

In the multivariate disease mapping, we can assume that, similarly to the univariate HBSM, the data are structured into spatial and non-spatial effects and can be graphically represented by the same four levels. The first level represents the local variability within each spatial unit as provided by the observed data assumed to have a Poisson distribution:

$$ \left.{O}_{ij}\right|{\theta}_{ij}\sim Poisson\left({E}_{ij}{\theta}_{ij}\right) $$

*O*_*ij*_ being the number of cases, *θ*_*ij*_ the relative risk, and *E*_*ij*_ the expected number of cases, all in a given area *i* and for disease *j*.

The main difference with the univariate disease mapping concerns the second level that represents the variability between spatial units and assumes a plausible dependence between diseases [34]. The a priori assumption considers that $\mathcal{U}$ or $\mathcal{V}$ follow a multivariate normal distribution (MVN) [35], that can be identified by *bb* and analytically written: *b* ~ *MVN*(0, Σ_*b*_), where Σ_*b*_ denotes the variance of parameter also known as the between-disease covariance matrix. To provide the log relative risks *θ*_*ij*_, Martinez-Beneito et al. [20] have recently proposed two M-based models that unified the multivariate disease mapping by considering Σ_*b*_ = *M* ^⊺^ *M*. One M-based model considers *M* as a fixed effects; the other considers them as random effects of the correlations between the diseases. The model can be written: log(*θ*_*ij*_) = Φ*M* where Φ is the matrix that contains the distribution of the underlying spatial patterns. For disease *j* and area *i*, log(*θ*_*j*_) = Φ_1_ *M*_1*j*_ + … + Φ_*i*_ *M*_*ij*_, where *M*_*ij*_ is the entry in *M*. When Φ follows three independent proper CAR distributions with different parameters, the M-based model is equal to the MCAR model proposed by Jin et al. [36] and equal to that of Gelfand and Vounatsou [37] in the case of two diseases. For brevity, we have applied a BYM spatial structure on four underlying factors (four diseases). This multivariate BYM model [26] assumes the presence of eight underlying patterns, four of them with spatially heterogeneous distributions and four with CAR distributions.

Finally, we compared the DICs of the independent Bayesian modeling in each cancer type with those of the multivariate BYM models to check the usefulness of the multivariate approach.

### Statistical software programs

Moran and Potthoff-Whittinghill statistics were computed using package DCluster of R 3.0.2. EBI was computed using package spdep of R [38]. The proximity-weighted matrix *w*_*ij*_ = exp(−*d*_*ij*_), a negative exponential function for exponential decay, was used in all three methods [9, 39, 40]. For the SpODT, we used version 0.9 of SpODT package [12, 27]. For SaTScan, we used SaTScan version 9.3 [11]. For HBSM and Poisson regression, we used WinBUGS version 1.4.3 to estimate the Bayesian parameters. More specifically, we called WinBUGS from R using package R2WinBUGS 2.1-19 [41]. For each model, we ran three chains. A burn-in of 60 000 iterations was performed and the posterior distribution was obtained using a sample of 20 000 iterations. Convergence was monitored graphically using time series plots checked with Monte-Carlo standard error [4].

## Results

**Fig. 1:**
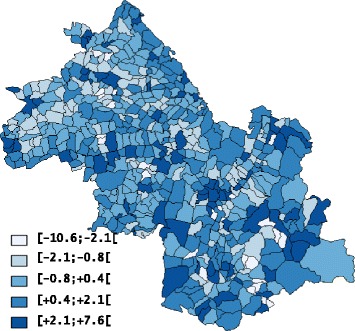
Choropleth map of the Townsend index of socioeconomic status in Département Isère according to the quintiles of its distribution (light and dark blue for low and high levels of deprivation, respectively)

In 2007, the population of Département Isère was 1,178,701 inhabitants. In 2007 too, among the 533 studied Communes, 55.16 % had fewer than one thousand inhabitants, 33.58 % had 1000 to 4000 inhabitants, and 11.26 % had more than 4000 inhabitants. In these Communes, 3898 cases of lung cancer, 8403 cases of prostate cancer, 3084 cases of colon-rectum cancer, and 1247 cases of bladder cancer were diagnosed between 1998 and 2007. In Département Isère, the median Townsend index was -0.002 (range: -10.5 to +7.6, Fig. 1).

### Results of the univariate analysis

#### Lung cancer

**Fig. 2:**
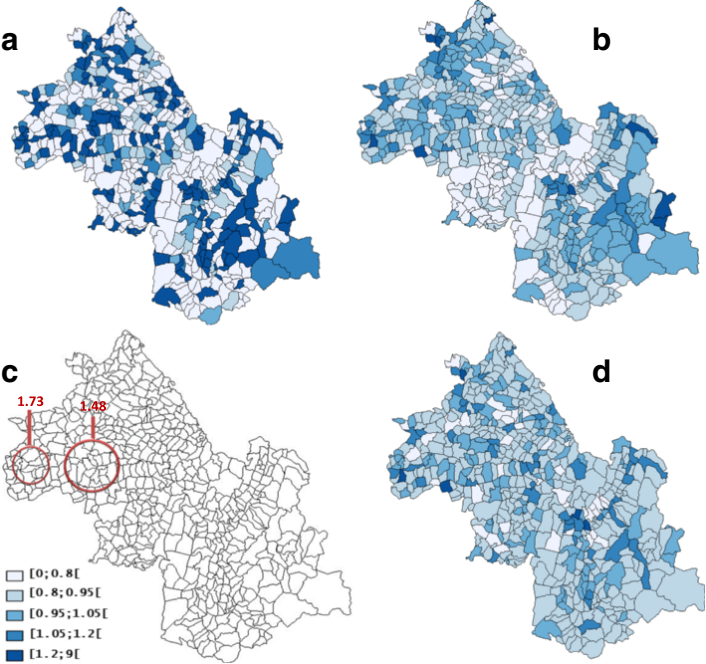
Clusters of lung cancer cases found by different methods: **a** geographic variations of standardized incidence ratio, **b** Mapping of the log relative risks by the CAR model using hierarchical Bayesian spatial modeling without adjustment on the Townsend index of socioeconomic status, **c** SaTScan clusters of high risk without adjustment on the Townsend index, **d** Mapping of the log relative risks by the heterogeneity model using hierarchical Bayesian spatial modeling without adjustment on the Townsend index. Four risk levels were considered (lightest to darkest color)

Spatial heterogeneity (*p* = 0.001) without “naive” spatial autocorrelation (*I* = 0.001, *p* = 0.874) or “true” spatial autocorrelation (EBI = 0.01, *p* = 0.019) was found for lung cancer (Table 1). Used without covariate, SpODT algorithm did not detect significant clusters of lung cancer (*p* = 0.5); however, SaTScan algorithm detected two significant clusters (RR = 1.48, *p* < 0.0001 and RR = 1.73, *p* < 0.001) (Fig. 2c). The Poisson model had the greatest DIC, which shows an over-dispersion of cases (Tables 2 and 3). The best Bayesian model applied to lung cancer data without covariate was the BYM model (Additional file 1: Figure S1a). The empirical variance of the $\mathcal{U}$ component was larger than that of the spatial heterogeneity $\mathcal{V}$ (Table 2).

**Table 1:** “Naive” spatial autocorrelation (Moran I), “true” Spatial autocorrelation (EBI), and heterogeneity (Potthoff-Whittinghill) test results

Cancer	Moran I	EBI	Potthoff-Whittinghill
Lung	0.001 (0.874)	0.01 (0.019)	(0.001)
Prostate	0.01 (0.001)	0.02 (0.001)	(0.001)
Colon-rectum	0.0004 (0.169)	0.007 (0.446)	(0.247)
Bladder	0.001 (0.346)	0.007 (0.05)	(0.003)

The results are expressed as: test statistic (*p*-value)

**Table 2:** Deviance information criteria and empirical variances of the Bayesian models (with their 95 % credible intervals, CI)

	Deviance information criteria				Empirical variances (95 % CI)	
Cancer	α(+βχ_ι_)	α + V_i_(+βχ_ι_)	α + U_i_(+βχ_ι_)	α + U_i_ + V_i_(+βχ_ι_)	s_u_^2^	s_v_^2^
Without covariate						
Lung	2172.8	2071.2	2082.8	2068.3	0.031 (0.004; 0.498)	0.057 (0.031; 0.085)
Prostate	2765.3	2631.4	2585.4	2589.2	0.008 (0.003; 0.019)	0.617 (0.310; 0.998)
Colon-rectum	1929.0	1931.0	1930.3	1932.5	0.0091 (0.002; 0.022)	0.0354 (0.007; 0.180)
Bladder	1374.6	1373.2	1371.0	1371.4	0.018 (0.005; 0.052)	0.241 (0.025; 1.015)
With covariate						
Lung	2016.5	2007.2	2009.9	2007.9	0.066 (0.013; 0.374)	0.011 (0.002; 0.030)
Prostate	2736.4	2630.2	2585.7	2588.5	0.007 (0.017; 0.002)	0.577 (0.261; 0.981)
Colon-rectum	1930.7	1933.1	1932.0	1933.3	0.007 (0.002; 0.024)	0.006 (0.002; 0.143)
Bladder	1368.3	1371.0	1367.4	1370.3	0.017 (0.0035; 0.056)	0.195 (0.030; 2.529)

(+***βχ***_***ι***_) in case of additional covariate - $\mathbf{\mathcal{U}}$ **:** Autocorrelation - $\mathbf{\mathcal{V}}$ **:** heterogeneity - ***s***_***u***_^2^: Autocorrelation empirical variance - ***s***_***v***_^2^: Heterogeneity empirical variance – $\mathbf{\mathcal{X}}$ Townsend index components

**Table 3:** The method of spatial analysis: summary on heterogeneity, clustering, and high-risk clusters

Cancer	Potthoff-Whittinghill	Moran I	EBI	Poisson	SpODT	SATSCAN	HBSM
Without covariate							
Lung	Heterogeneity	No clustering	Clustering	No clustering	No cluster	Two clusters	Heterogeneity and clustering
Prostate	Heterogeneity	Clustering	Clustering	No clustering	Six clusters	Five clusters	Only clustering
Colon rectum	No heterogeneity	No clustering	No clustering	No clustering	No cluster	Two clusters	No heterogeneity and no clustering
Bladder	Heterogeneity	No clustering	Clustering	No clustering	One cluster	No cluster	Only clustering
With covariate							
Lung	---	---		No clustering	No cluster	No cluster	Only heterogeneity
Prostate	---	---		No clustering	Five clusters	No cluster	Only clustering
Colon rectum	---	---		No clustering	No cluster	No cluster	No heterogeneity and no clustering
Bladder	---	---		No clustering	One cluster	No cluster	Only clustering

With the Townsend index of socioeconomic status as covariate, SpODT and SaTScan algorithms did not detect significant high-risk clusters. The DICs and variances *s*_*v*_^2^ and *s*_*u*_^2^ of the Bayesian models (RR >1.05) are shown in Table 2. The use of the Townsend index decreased sharply the DIC as well as variances *s*_*v*_^2^ and *s*_*u*_^2^ (Table 2). The socioeconomic inequalities seemed to increase the spatial variations of lung cancer incidence (*β* = 0.067 [0.054; 0.080]) (Table 4).

**Table 4:** The method of spatial analysis: effect of the socioeconomic status on the spatial structure of cancer incidence [regression coefficient with 95 % confidence intervals for Poisson models or credible intervals for HBSM]

	SpODT	SATSCAN	HBSM			
			α + βχ_ι_	α + V_i_ + βχ_ι_	α + U_i_ + βχ_ι_	α + U_i_ + V_i_ + βχ_ι_
Lung	---	---	0.059 [0.050; 0.069]	0.067 [0.054; 0.080]^a^	0.065 [0.053; 0.077]	0.068 [0.055; 0.080]
Prostate	5 clusters	---	-0.017 [-0.023; -0.011]	-0.010 [-0.021; -0.0005]	-0.012 [-0.022; -0.001]^a^	-0.011 [-0.021; -0.0009]
Colon rectum	---	---	-0.001 [-0.011; 0.009]^a^	0.0003 [-0.011; 0.012]	0.0004 [-0.010; 0.011]	0.0007 [-0.011; 0.013]
Bladder	1 cluster	---	0.023 [0.007; 0.039]	0.023 [0.004; 0.043]	0.022 [0.003; 0.041]^a^	0.024 [0.003; 0.045]

^a^Best Bayesian model in term of DIC, βχ_*ι*_ additional covariate - $\mathbf{\mathcal{U}}$ **:** Autocorrelation - $\mathbf{\mathcal{V}}$ **:** heterogeneity $\mathbf{\mathcal{X}}$ Townsend index components

#### Prostate cancer

**Fig. 3:**
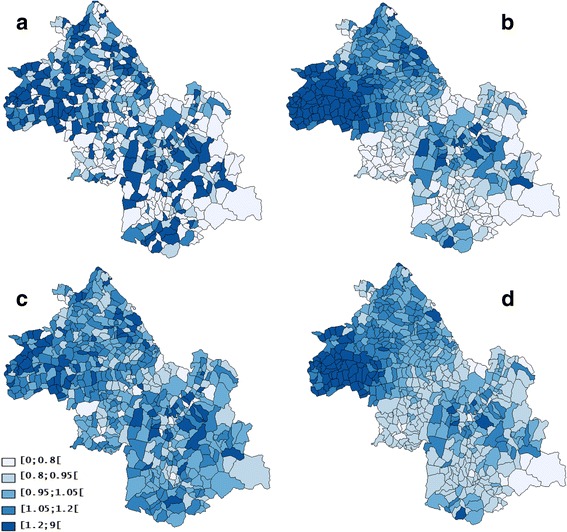
Clusters of prostate cancer cases found by different methods: **a** SpODT clusters without adjustment on the Townsend index, **b** SaTScan clusters without adjustment on the Townsend index, **c** Mapping of the log relative risks estimated by BYM model using hierarchical Bayesian spatial modeling without adjustment on the Townsend index. Four risk levels were considered (lightest to darkest color), **d** SpODT clusters with adjustment on the Townsend index

A Spatial heterogeneity (*p* = 0.001) with “naive” autocorrelation (*I* = 0.01, *p* = 0.001) and “true” autocorrelation (EBI = 0.02, *p* = 0.001) were found with prostate cancer data (Table 1). The SpODT algorithm cut out the geographical area into eleven zones with different risk levels (*p* < 0.0001). The mapping of these zones identified six high-risk clusters located at the boundaries of the study area; precisely, four clusters in the Northwestern part (SIRs = 1.68, 1.39, 1.14, and 1.12), a fifth at the Southern part (SIR = 1.16), and a sixth in the Northern part (SIR = 1.04) (Fig. 3a).

**Fig. 4:**
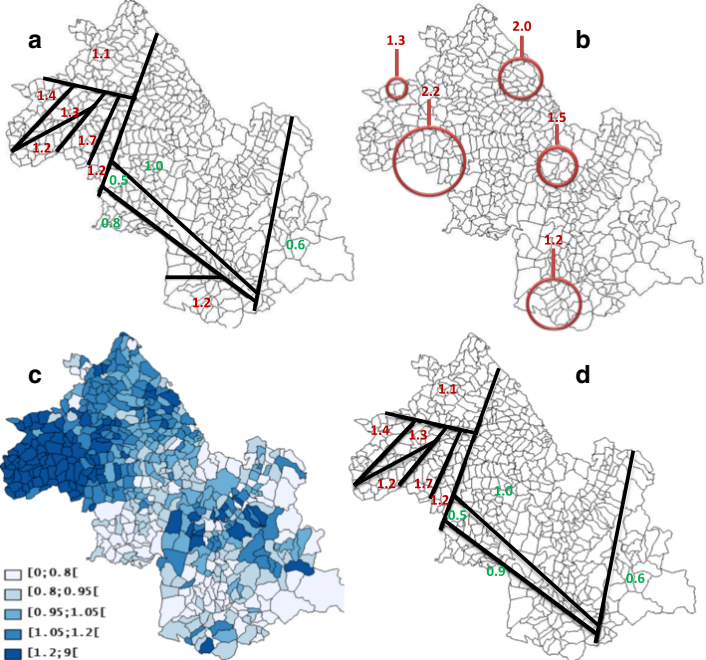
Mapping of prostate cancer: **a** geographic variations of standardized incidence ratio, **b** Mapping of the log relative risks estimated by CAR model using hierarchical Bayesian spatial modeling without adjustment on the Townsend index, **c** Mapping of the log relative risks estimated by heterogeneity model using hierarchical Bayesian spatial modeling without adjustment on the Townsend index, **d** Mapping of the log relative risks estimated by CAR model using hierarchical Bayesian spatial modeling with adjustment on the Townsend index. Four risk levels were considered (lightest to darkest color)

SaTScan located a main cluster of prostate cancer cases at the center of the area (RR = 1.51, *p* < 0.0001). In addition, four other significant clusters were located at the boundaries of the study area (RR = 1.31, *p* < 0.0001; RR = 2.16, *p* < 0.0001; RR = 1.20, *p* < 0.0001; and RR = 1.98, *p* < 0.01) (Fig. 3b) The Poisson and the constant risk models had the highest DICs, which reveal an over-dispersion of cases (Tables 2 and 3). The CAR model was the best among the Bayesian approaches (Table 2). Unlike the heterogeneity model (Fig. 4c), mapping the relative risk according to BYM (Fig. 3c) and CAR (Fig. 4b) models showed an over-incidence (RR >1.2) of prostate cancer and clusters in the study area.

With the Townsend index as covariate, SaTScan detected no clusters whereas SpODT located five clusters (*p* < 0.005) in the Northwest of the area; it merged only the clusters previously found in the Northwestern and Southern parts of the study area (SIR = 0.93) (Fig. 3d). There was also no change in the DIC and variances *s*_*v*_^2^ and *s*_*u*_^2^ remained stable. The CAR model showed that the socioeconomic inequalities had a slight impact on the spatial variations of prostate cancer (*β* = -0.012 [-0.022; -0.001]) (Table 4).

#### Colon-rectum cancer

**Fig. 5:**
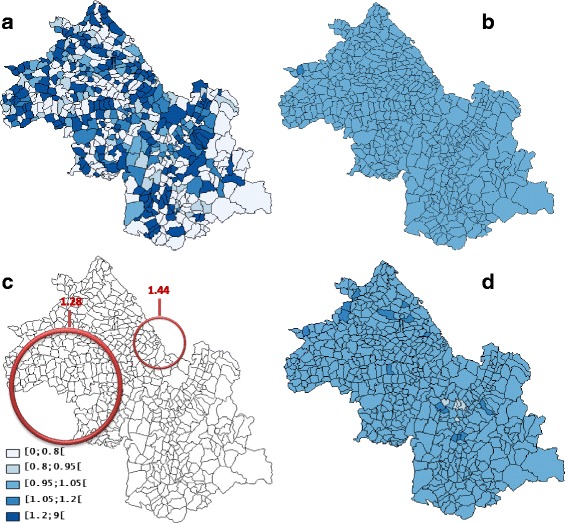
Clusters of colon-rectum cancer cases found by different methods: **a** geographic variations of standardized incidence ratio, **b** Mapping of the log relative risks estimated by the CAR model using hierarchical Bayesian spatial modeling without adjustment on the Townsend index, **c** SaTScan clusters without adjustment on the Townsend index (2 clusters of high risk), **d** Mapping of the log relative risks estimated by the heterogeneity model using hierarchical Bayesian spatial modeling without adjustment on the Townsend index (lightest to darkest color)

The analysis found neither heterogeneity nor spatial autocorrelation (Table 1). SpODT detected no significant clusters (*p* = 0.19). SaTScan detected two clusters (RR = 1.28, *p* < 0.001 and RR = 1.44, *p* < 0.001) (Fig. 5c). The Poisson and all the Bayesian models had the same DIC, which shows a homogeneous distribution of colon-rectum cancer cases (Table 2). Mapping the relative risk according to CAR model (Fig. 5b; Additional files 1–6) showed a homogeneous risk over the study area.

With the Townsend index, neither SpODT nor SaTScan could detect significant clusters and the results of Bayesian modeling did not change (Table 3). With the Bayesian model, the credible intervals and variances *s*_*v*_^2^ and *s*_*u*_^2^ were stable but large. The constant risk model was the best Bayesian model in terms of DIC (Table 2). The socioeconomic inequalities did not seem to affect the spatial variations of colon-rectum cancer incidence in Département Isère (*β* = -0.001 [-0.011; 0.009]) (Table 4).

#### Bladder cancer

Spatial heterogeneity (*p* < 0.001) without “naive” autocorrelation (*I* = 0.001; *p* = 0.346) and “true” autocorrelation (*I* = 0.007; *p* = 0.05) were found for bladder cancer (Table 1). SpODT detected starred clusters in the Centre-East of the area (RR = 1.44, *p* < 0.01). SaTScan detected no clusters.

**Fig. 6:**
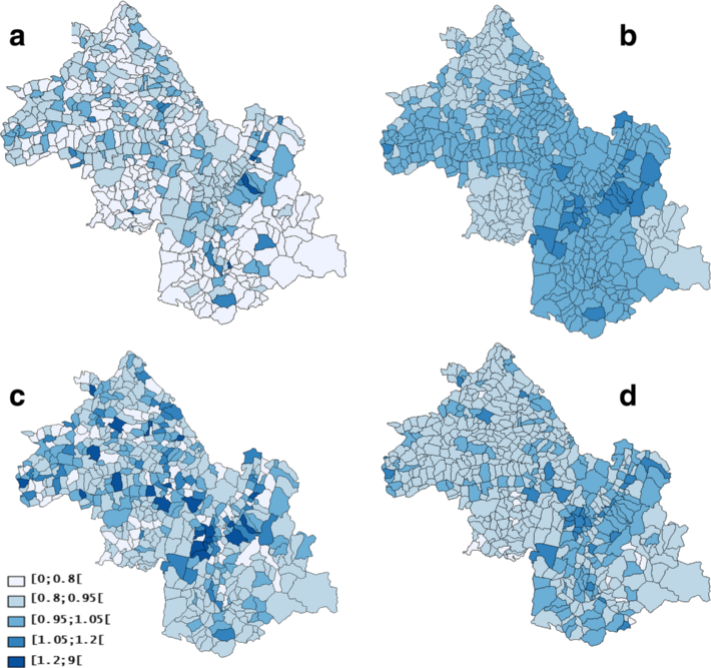
Clusters of bladder cancer cases found by different methods: **a** geographic variations of standardized incidence ratio, **b** Mapping of the log relative risks estimated by the CAR model using hierarchical Bayesian spatial modeling without adjustment on the Townsend index, **c** Mapping of the log relative risks estimated by the heterogeneity model using hierarchical Bayesian spatial modeling without adjustment on the Townsend index, **d** Mapping of the log relative risks estimated by the CAR model using hierarchical Bayesian spatial modeling with adjustment on the Townsend index

Taking into account the spatial structure of bladder cancer cases, the best Bayesian model in terms of DIC was the CAR model (Table 2; Fig. 6b). With the Townsend index, SpODT detected a shrunk cluster. The introduction of this index into the best Bayesian model did not change a lot the DIC (Tables 2 and 3). In the BYM models, variance *s*_*v*_^2^ was greater than variance *s*_*u*_^2^ (Table 2). Mapping the relative risk according to CAR model with adjustment on the Townsend index showed that the socioeconomic inequalities had an impact on the spatial variations of bladder cancer incidence in Département Isère (*β* = 0.022 [0.003; 0.041]) (Fig. 6d; Table 4).

### Results of the multivariate analysis

Finally, the DICs of independent disease modeling, with or without covariate, were not higher than those of the multivariate BYM models. The multivariate BYM model with fixed effects was the best model (Table 5). The correlations between the effects of these cancers were very important in the case of the pair lung-bladder cancers (Table 6). The mapping of the diseases led to less smoothing than with the univariate BYM models. However, mapping the relative risk with the multivariate approach showed no longer the clusters, not even those of prostate cancer cases (Additional files 1–6).

**Table 5:** Deviance information criteria for independent modeling and multivariate modeling (M-based BYM) of the Bayesian models

	Deviance information criteria					
Modeling	*α*(+*βχ*_*ι*_)	$\alpha +{\mathbf{\mathcal{V}}}_i\left(+\beta {\chi}_{\iota}\right)$	$\alpha +{\mathbf{\mathcal{U}}}_i\left(+\beta {\chi}_{\iota}\right)$	$\alpha +{\mathbf{\mathcal{U}}}_i+{\mathbf{\mathcal{V}}}_i\left(+\beta {\chi}_{\iota}\right)$	M-based BYM with fixed effects	M-based BYM with random effects
Without covariate	8141.7	8006.8	7969.5	7961.4	6947.84	7131.05
With covariate	8051.9	7941.5	7895	7900		

(+***βχ***_***ι***_) in case of additional covariate - $\mathbf{\mathcal{U}}$ **:** Autocorrelation - $\mathbf{\mathcal{V}}$ **:** heterogeneity $\mathbf{\mathcal{X}}$ Townsend index components

**Table 6:** Posterior means of the between-disease correlation matrix for the M-based BYM model with fixed effects

Cancer	Lung	Prostate	Bladder	Colon-rectum
Lung	1			
Prostate	0.03	1		
Bladder	0.60	0.29	1	
Colon-rectum	0.40	0.14	0.39	1

## Discussion

Different methods of spatial analysis suitable for cluster detection and epidemiological monitoring in small areas were used here to: i) describe spatial heterogeneity and autocorrelation; ii) evaluate the impact of heterogeneity on global spatial autocorrelation; and: iii) search for an effect of the socioeconomic status on geographical differences in cancer incidence by analyzing the overall spatial structure or detecting high-risk areas. More precisely, the work aimed at examining whether deprivation is an explanatory or a confusion factor of the spatial distribution of some cancers. This study highlights the importance of using both global [42] and local methods of cluster detection taking into account heterogeneity [43]. “Naive” spatial autocorrelation and heterogeneity were found only with prostate cancer data. The adjusted Moran’s I method [43] detected mainly a spatial autocorrelation in lung and bladder cancer as well as in prostate cancer by taking into account the spatial heterogeneity. In all cancers, “true” Moran’s I value was greater than “naive” Moran I value. This shows that it is important to include small spatial units in the calculation of spatial units in the calculation of spatial test statistics to be able to detect spatial autocorrelation.

SpODT is an approach recently applied to spatial distribution of cancer risk. Like SaTScan, one advantage of SpODT is its ability to overcome the administrative boundaries; another is that its implementation does not require the use of a proximity matrix, which avoids the problems related to the choice of this matrix (as in HBSM) [12]. According to the algorithm stopping criteria, these two methods require sensitivity analyses. With SaTScan, the optimal clusters were found after sensitivity analyses regarding the size of the window. In the previous literature, few authors have mentioned these sensitivity analyses or the search for optimal parameters. Depending on the user settings, the lack of a sensitivity analysis is not in favor of a method’s reproducibility [44]. In disease mapping, Bayesian smoothing remains important because it allows taking into account spatial and non-spatial effects in risk estimation [1, 4, 45]. In small-area studies, problems of robustness of the estimates can be overcome by the use of hierarchical Bayesian spatial modeling; this warrants a better understanding of the risk levels in spatial epidemiology. In a simulation study, Aamodt et al. [32] have shown that BYM model is better than SaTScan for local cluster detection in case of high relative risks [46]. Bayesian models allow both global and local detection through criteria such as the variance of the autocorrelation, heterogeneity components, and DIC. The multivariate disease mapping approach through joint modeling [15, 34, 36, 37] provides also a considerable improvement of spatial analysis by including information on correlations between diseases and by reducing smoothing effects. The present study shows the specificities of each method that will be discussed according to the results by cancer type.

The case of colon-rectum cancer (where global methods found neither heterogeneity nor spatial autocorrelation) allowed an evaluation of local clustering methods. In this case, the use of a spatial model is superfluous; indeed, all the methods agreed on the absence of clusters of colon-rectum cancers. Only SaTScan detected clusters of high risk with RRs <1.5 when the analysis was carried out without covariate. Guttmann et al. [7] have shown in a simulation that the performance of SaTScan increased with the size of the population. Likewise, in small areas when Commune is a proxy for patient exact location, our results corroborate those of Lemke et al., Jeffery et al., and Ozonoff et al. [32, 47, 48]. These studies demonstrated that the power of detecting clusters with SaTScan decreased together with the level of spatial resolution. The shape of the cluster was also discussed by Goujon-Bellec et al. [49] who found that the elliptic scan method seems more appropriate than the circular scan method in detecting clusters of rare diseases over large regions. With simulation studies, other authors, such Aamodt et al. [46] have found that SaTScan was more efficient than BYM model in detecting clusters with relatively low relative risks. This was corroborated in colon-rectum cancer. The HBSM confirmed its ability to detect a homogeneous risk with colon-rectum cancer and seemed to be less affected by population size, spatial resolution, or cluster shape. Furthermore, the use of an additional covariate (here, the Townsend index) reduced greatly the performance of SaTScan in terms of specificity [50].

In the case of prostate cancer, all the methods converged to the same conclusion. The global clustering methods found a spatial autocorrelation and a spatial heterogeneity and all the local methods showed coherent clusters. SaTScan failed to detect an effect of the socioeconomic status. SpODT as well as the univariate HBSM detected coherent clusters. Our results with prostate cancer data raised the problem of edge effects in local cluster detection as previously found by Johnson [17]. An edge effect can be defined as an impact on the results of features specific to the boundaries of the study area, such as spatial censoring. Precisely, some subjects may not be observed because they are out of the study area and thus excluded from the spatial analysis [4]. Indeed, the cluster of prostate cancer cases detected by SaTScan in the center of the area is probably erroneous because other clusters were also located at the boundaries of that area. Actually, Guttmann et al. [42] have shown that false clusters are numerous when edge effects are important. To correct these effects, the area under sensitivity analyses may be extended to other neighboring areas (here, an extension from Département Isère to the whole Rhône-Alpes Region). The use of more homogeneous spatial units than the current Communes, such as the French “Ilots Regroupés pour l’Information Statistique” (IRIS), may also eliminate or reduce the edge effects [51]. Little and Rubin have also proposed to solve this problems by the use of methods that consider the external areas as missing data [4]. We may mention here that SpODT was able to detect more precise clusters than SaTScan, especially when the Townsend index was taken into account in presence of autocorrelation. Poisson model and HBSM found that larger socioeconomic inequalities decreased the incidence of prostate cancer. In fact, deprived patients are often diagnosed at symptomatic stages, a fact that has been precisely detected by SpODT in the Southwestern part of Isère. This should be kept in mind because, in deprived people, other cancers, such as skin melanoma and breast cancer are often diagnosed at advanced stages [52].

In the cases of lung and bladder cancers, EBI showed “true” spatial autocorrelation while Moran’s I test failed to find autocorrelation. These results highlight the importance of taking into account the heterogeneity in small areas when attempting to identify the spatial pattern of a disease. Contrarily to SaTScan, SpODT did not find clusters of lung cancer. Lung cancer results showed that a lower DIC (with vs. without introducing the Townsend index into the model) has identified an effect for that index on the geographical variations of the incidence in terms of spatial heterogeneity. In lung cancer, the Townsend index influenced greatly the randomcomponent whereas, in the bladder cancer, it was spatial autocorrelation that influenced the spatial analysis. The Poisson model and the univariate HBSM coefficient have shown that, in these two cancers, the incidence increases together with the socioeconomic inequalities. In the specific case of lung cancer, the socioeconomic status seemed to be a surrogate for various lifestyle factors (e.g., alcohol/tobacco consumption). Thus, as in previous studies, the socioeconomic status should not be overlooked, as a risk factor, in examining lung cancer etiology [53]. One, now classical observation, is that bladder cancer shares common risk factors with lung cancer (e.g., tobacco consumption). This was shown by Cassetti and al. [18] in a spatial study in Umbria, Italy. The multivariate modeling found also a correlation between these two cancers (posterior mean estimation: 0.6). In terms of DIC, the multivariate BYM model was the best model. These results corroborate those of Martinez-Beneito [20] who recommended multivariate disease mapping models to epidemiologists interested in the spatial variations of several diseases. Changes in the DIC with HBSM may thus be used to identify the most credible spatial model vs. other competing spatial competing models and detect the cluster of high risk. Indeed, we have used the DIC on Colonna and Sauleau [5] updated data to choose the best univariate Bayesian model and found similar results. However, some covariates and spatial patterns may be mixed up with the random effects; their inclusion in a spatial analysis can lead to biased estimates of the fixed effects [54].

Using CAR models, some authors such as Reich et al. or Hughes and Haran [55, 56], advise the use of a model without confounding random effects even if its DIC is greater than that of the usual spatial model when the goal is to study the association between any covariate and the disease under study. Here, in all the approaches we used, we did not check the existence of spatial confounding.

### Limitations

In this empirical assessment of the efficacy of cluster detection methods, the results were consistent across all methods only in the case of prostate cancer. This raises questions in terms of power and precision of spatial cluster detection methods and suggests that power and precision would increase together with the event rate. However, checking both these hypotheses and assessing the efficacy of the discussed methods in other plausible epidemiological situations require analyses conducted in a systematic way. These limitations could be solved properly by simulation studies.

## Conclusion

The present methodological and comparative study on the performance of cluster detection methods in oncology was able to show the importance of using a variety of methods not only to find coherent spatial clusters but also to determine the influence of a given factor on the geographical distribution of cancer incidence. The study is a practical example of cluster identification in presence of heterogeneity and unknown common factors. It demonstrates that it is possible to obtain a quantitative estimation of the effect of the socioeconomic status on the differences in cancer incidence, especially through the Bayesian approaches able to integrate prior information. The multivariate spatial modeling is recommended to map several diseases and take into account their potential links.

## Additional files

Additional file 1: Figure S1. Clusters of lung cancer cases found by BYM models: (a) Mapping of the log relative risks estimated by the BYM model using hierarchical Bayesian spatial modeling without adjustment on the Townsend index, (b) Mapping of the log relative risks estimated by the BYM model using hierarchical Bayesian spatial modeling with adjustment on the Townsend index, (c) Mapping of the log relative risks estimated by the M-based BYM model using hierarchical Bayesian spatial modeling with fixed effects, (d) Mapping of the log relative risks estimated by the M-based BYM model using hierarchical Bayesian spatial modeling with random effects. (PDF 205 kb)
Additional file 2: Figure S2. Clusters of prostate cancer cases found by BYM models: (a) Mapping of the log relative risks estimated by the BYM model using hierarchical Bayesian spatial modeling without adjustment on the Townsend index, (b) Mapping of the log relative risks estimated by the BYM model using hierarchical Bayesian spatial modeling with adjustment on the Townsend index, (c) Mapping of the log relative risks estimated by the M-based BYM model using hierarchical Bayesian spatial modeling with fixed effects, (d) Mapping of the log relative risks estimated by the M-based BYM model using hierarchical Bayesian spatial modeling with random effects. (PDF 204 kb)
Additional file 3: Figure S3. Clusters of colon-rectum cancer cases found by BYM models: (a) Mapping of the log relative risks estimated by the BYM model using hierarchical Bayesian spatial modeling without adjustment on the Townsend index, (b) Mapping of the log relative risks estimated by the BYM model using hierarchical Bayesian spatial modeling with adjustment on the Townsend index, (c) Mapping of the log relative risks estimated by the M-based BYM model using hierarchical Bayesian spatial modeling with fixed effects, (d) Mapping of the log relative risks estimated by the M-based BYM model using hierarchical Bayesian spatial modeling with random effects. (PDF 206 kb)
Additional file 4: Figure S4. Clusters of bladder cancer cases found by BYM models: (a) Mapping of the log relative risks estimated by the BYM model using hierarchical Bayesian spatial modeling without adjustment on the Townsend index, (b) Mapping of the log relative risks estimated by the BYM model using hierarchical Bayesian spatial modeling with adjustment on the Townsend index, (c) Mapping of the log relative risks estimated by the M-based BYM model using hierarchical Bayesian spatial modeling with fixed effects, (d) Mapping of the log relative risks estimated by the M-based BYM model using hierarchical Bayesian spatial modeling with random effects. (PDF 214 kb)
Additional file 5: Figure S5. Clusters of colon-rectum cancer cases found by different methods with alternative cut-points of relative risks: (a) geographic variations of standardized incidence ratio, (b) Mapping of the log relative risks estimated by the CAR model using hierarchical Bayesian spatial modeling without adjustment on the Townsend index, (c) SaTScan clusters without adjustment on the Townsend index (2 clusters of high risk), (d) Mapping of the log relative risks estimated by the heterogeneity model using hierarchical Bayesian spatial modeling without adjustment on the Townsend index (lightest to darkest color). (PDF 367 kb)
Additional file 6: Table S1. Posterior means of the between-disease correlation matrix for the M-based BYM model with random effects. (DOCX 32 kb)

## Abbreviations

BYM: Besag-York-Mollié
CAR: Conditional autoregressive
CART: Classification and regression tree
DIC: Deviance information criterion
EBI: Empirical Bayes Index
GAM: Geographical analysis machine
HBSM: Hierarchical Bayesian spatial modeling
MCMC: Markov-chain Monte-Carlo
RR: Relative risk
SaTScan: Spatial scan statistic of Kulldorff
SIR: Standardized incidence ratio
SpODT: Spatial oblique decision Tree
