# Supplementary figures and images for: Impact of socioeconomic inequalities on geographic disparities in cancer incidence: comparison of methods for spatial disease mapping
Source: BMC Med Res Methodol. 2016 Oct 12;16:136. doi: 10.1186/s12874-016-0228-x (PMC5059978; doi:10.1186/s12874-016-0228-x)

**a**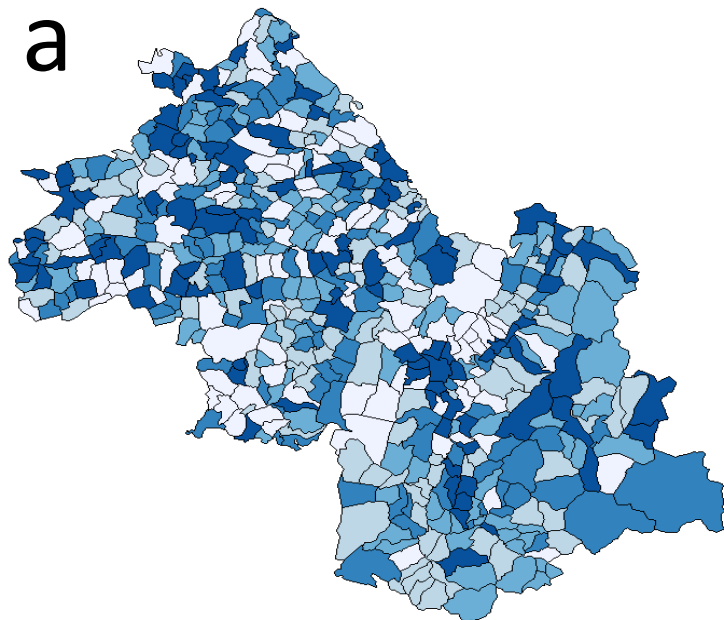**b**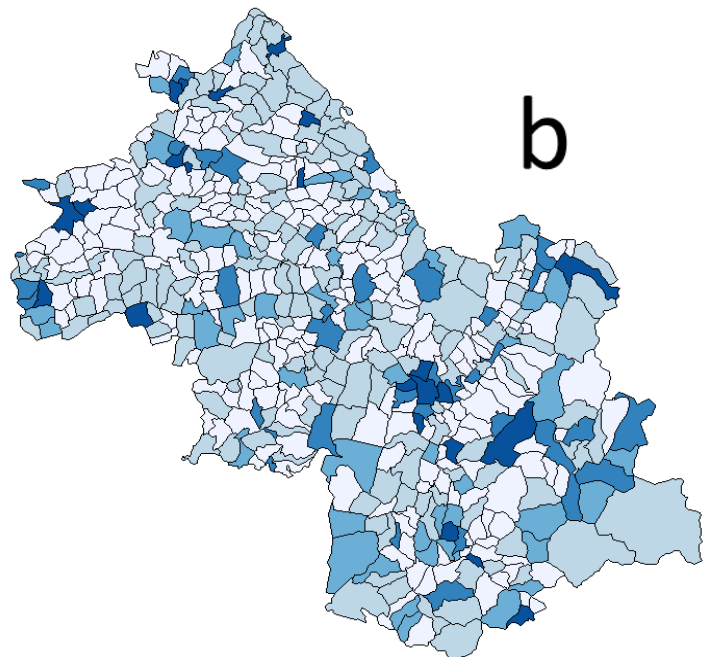**c**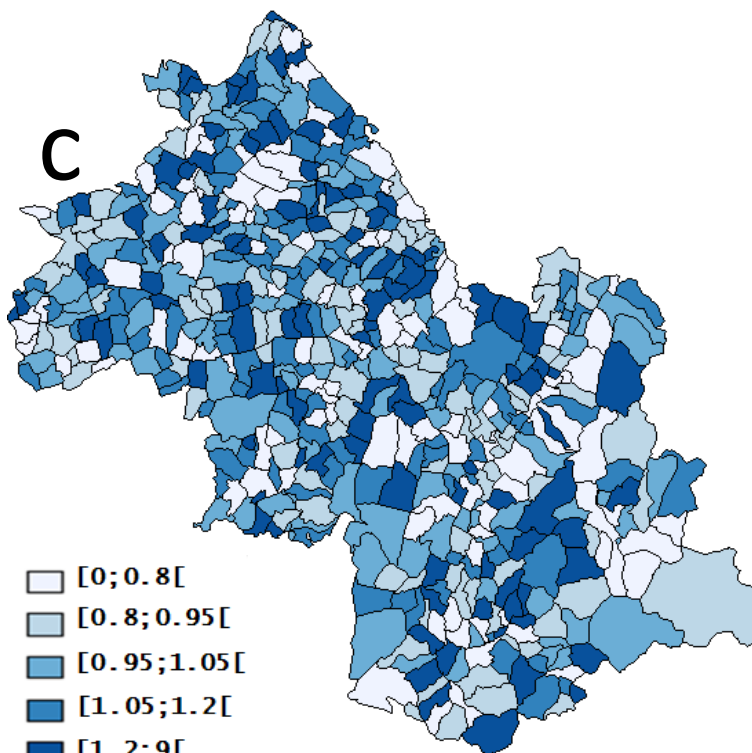**d**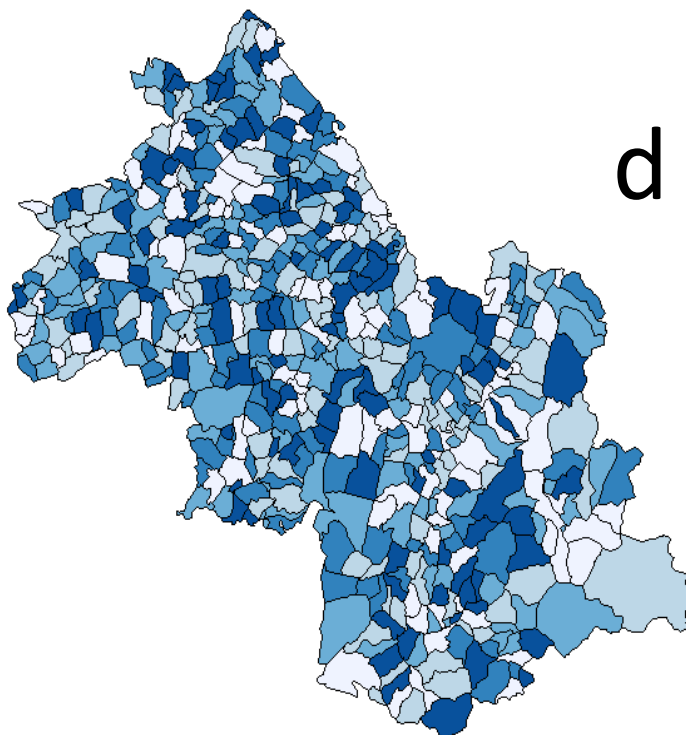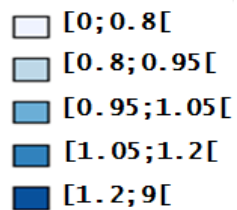

Supplement: Additional file 1: Figure S1. — Clusters of lung cancer cases found by BYM models: (a) Mapping of the log relative risks estimated by the BYM model using hierarchical Bayesian spatial modeling without adjustment on the Townsend index, (b) Mapping of the log relative risks estimated by the BYM model using hierarchical Bayesian spatial modeling with adjustment on the Townsend index, (c) Mapping of the log relative risks estimated by the M-based BYM model using hierarchical Bayesian spatial modeling with fixed effects, (d) Mapping of the log relative risks estimated by the M-based BYM model using hierarchical Bayesian spatial modeling with random effects. (PDF 205 kb) [file 12874_2016_228_MOESM1_ESM.pdf]

**a**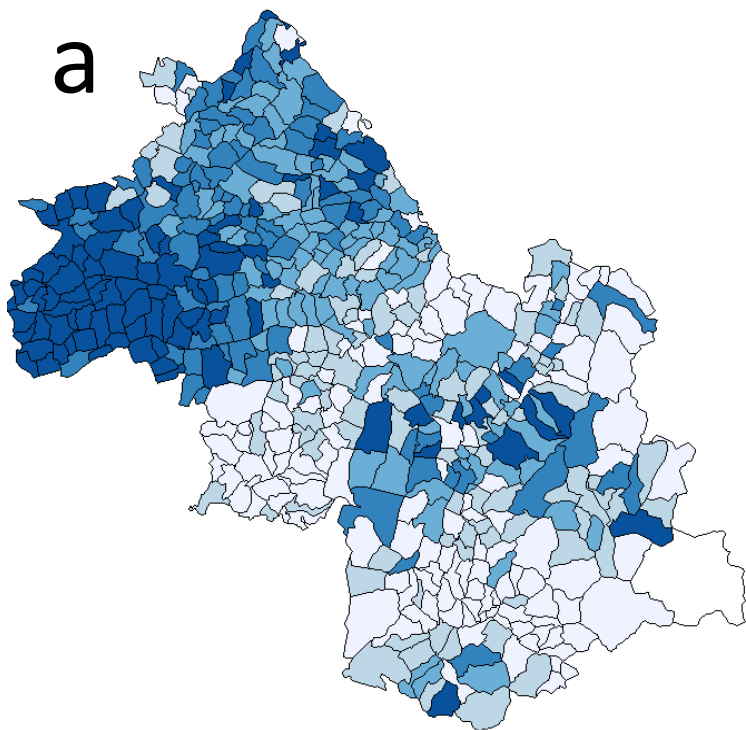**b**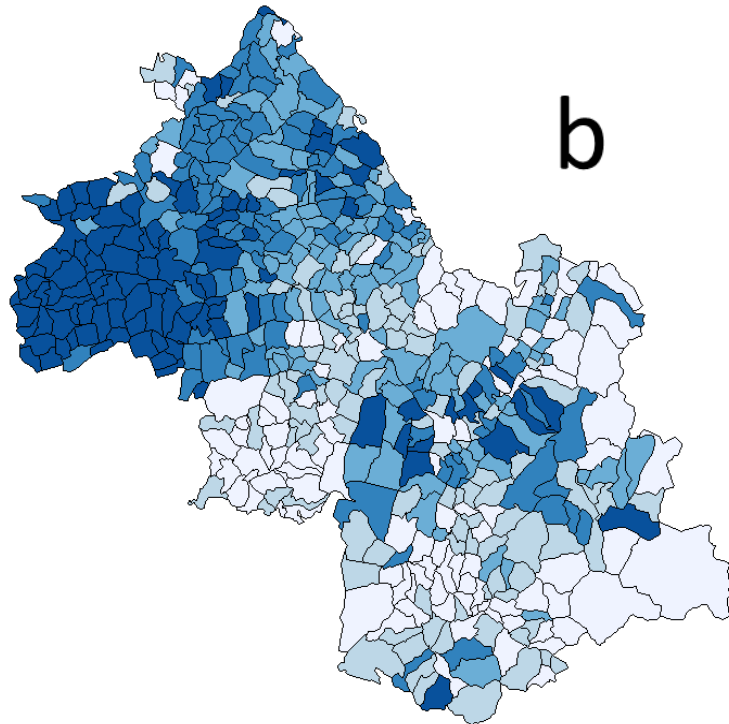**c**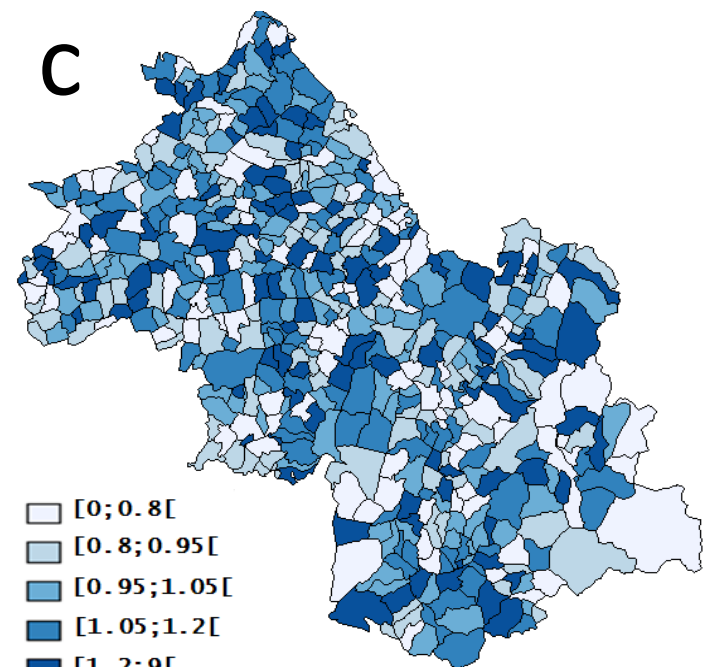**d**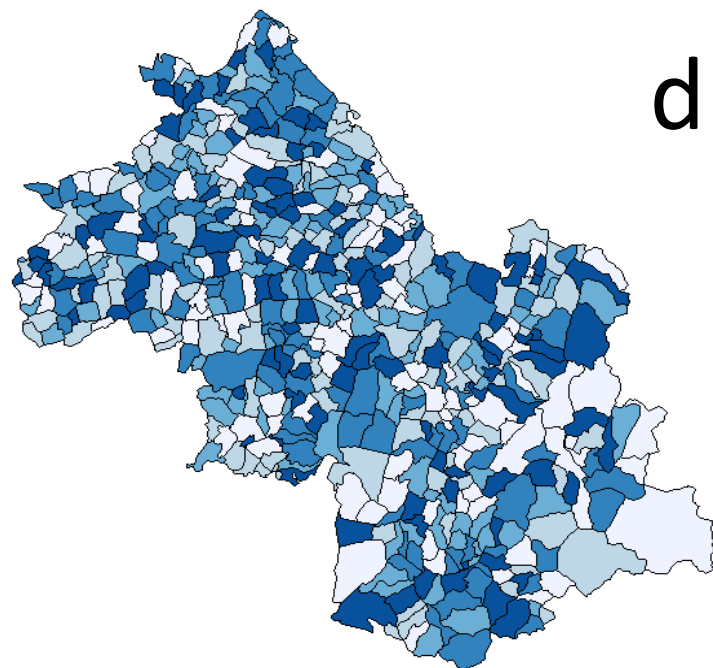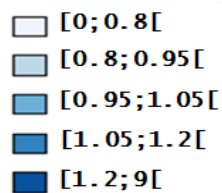

Supplement: Additional file 2: Figure S2. — Clusters of prostate cancer cases found by BYM models: (a) Mapping of the log relative risks estimated by the BYM model using hierarchical Bayesian spatial modeling without adjustment on the Townsend index, (b) Mapping of the log relative risks estimated by the BYM model using hierarchical Bayesian spatial modeling with adjustment on the Townsend index, (c) Mapping of the log relative risks estimated by the M-based BYM model using hierarchical Bayesian spatial modeling with fixed effects, (d) Mapping of the log relative risks estimated by the M-based BYM model using hierarchical Bayesian spatial modeling with random effects. (PDF 204 kb) [file 12874_2016_228_MOESM2_ESM.pdf]

**a**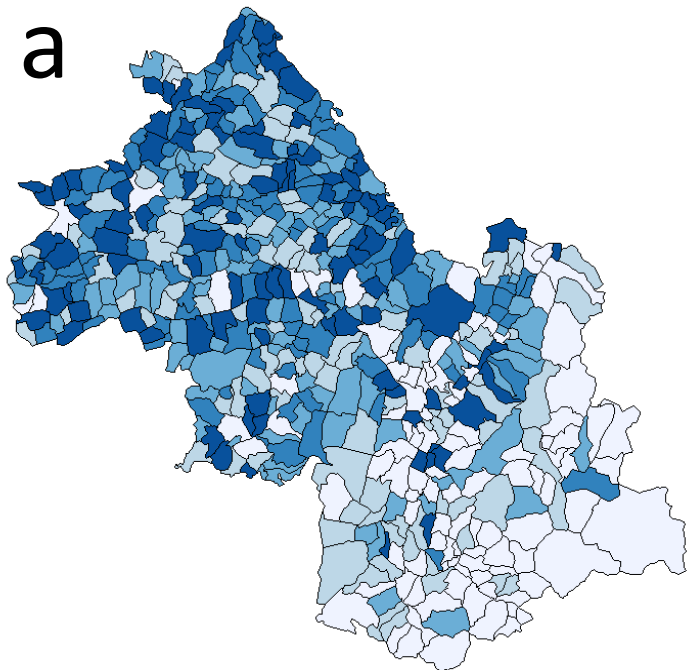**b**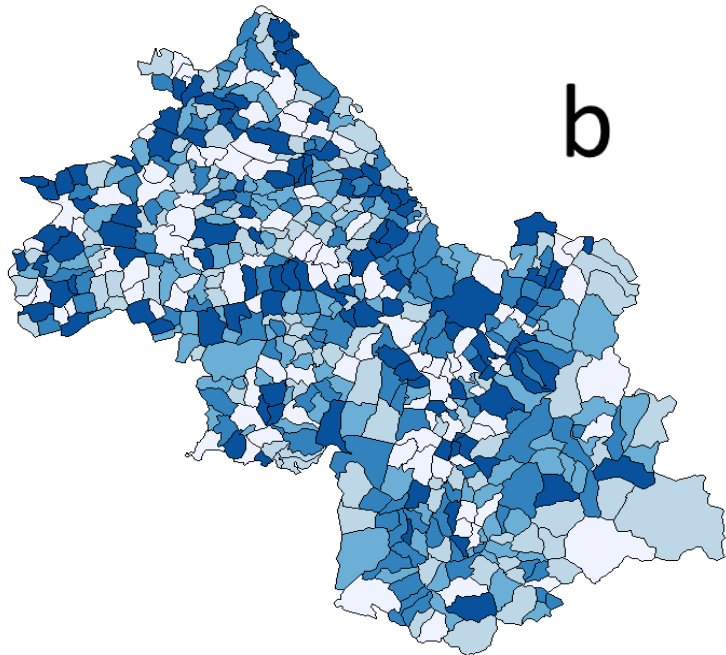**c**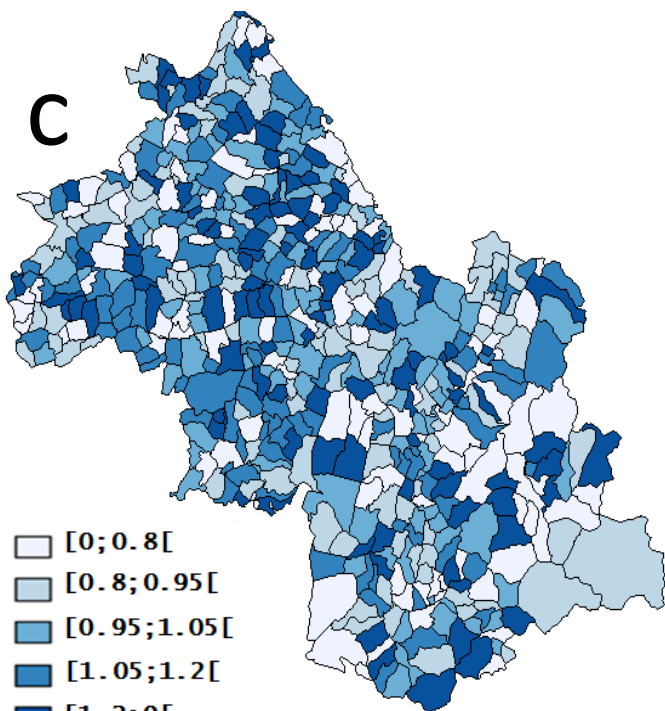**d**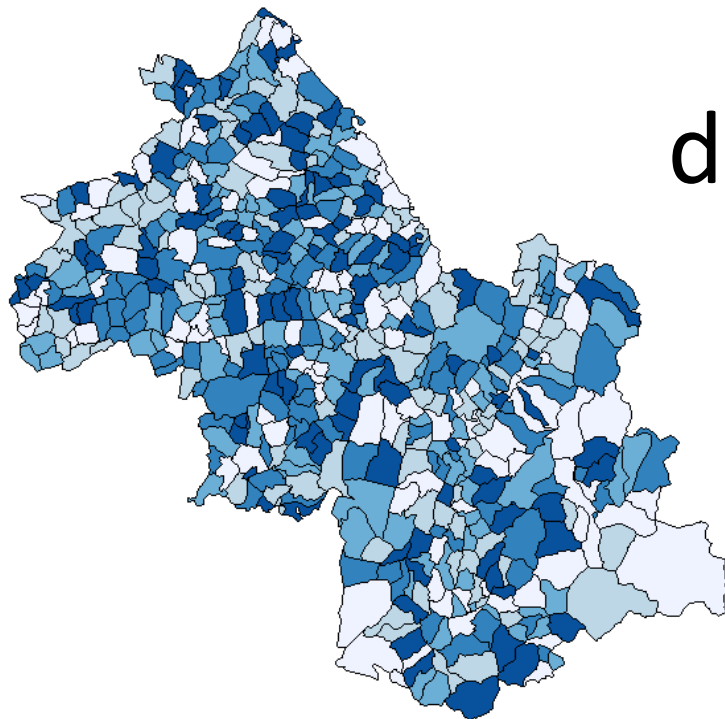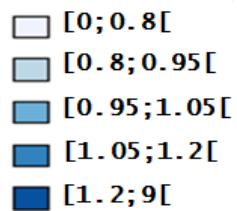

Supplement: Additional file 3: Figure S3. — Clusters of colon-rectum cancer cases found by BYM models: (a) Mapping of the log relative risks estimated by the BYM model using hierarchical Bayesian spatial modeling without adjustment on the Townsend index, (b) Mapping of the log relative risks estimated by the BYM model using hierarchical Bayesian spatial modeling with adjustment on the Townsend index, (c) Mapping of the log relative risks estimated by the M-based BYM model using hierarchical Bayesian spatial modeling with fixed effects, (d) Mapping of the log relative risks estimated by the M-based BYM model using hierarchical Bayesian spatial modeling with random effects. (PDF 206 kb) [file 12874_2016_228_MOESM3_ESM.pdf]

**a**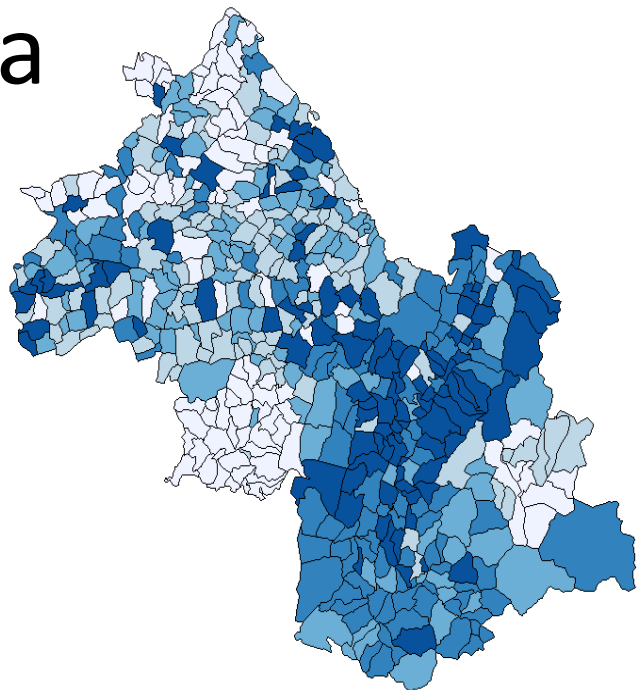**b**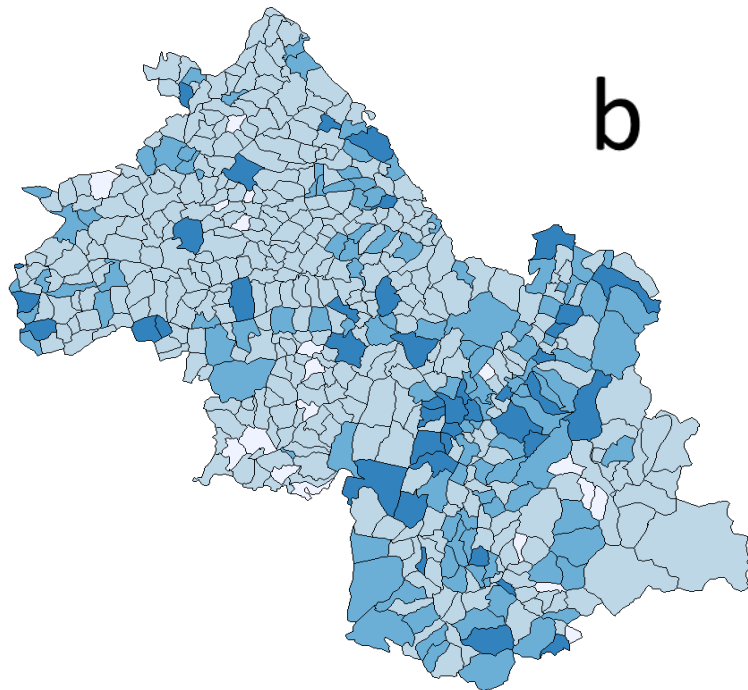**c**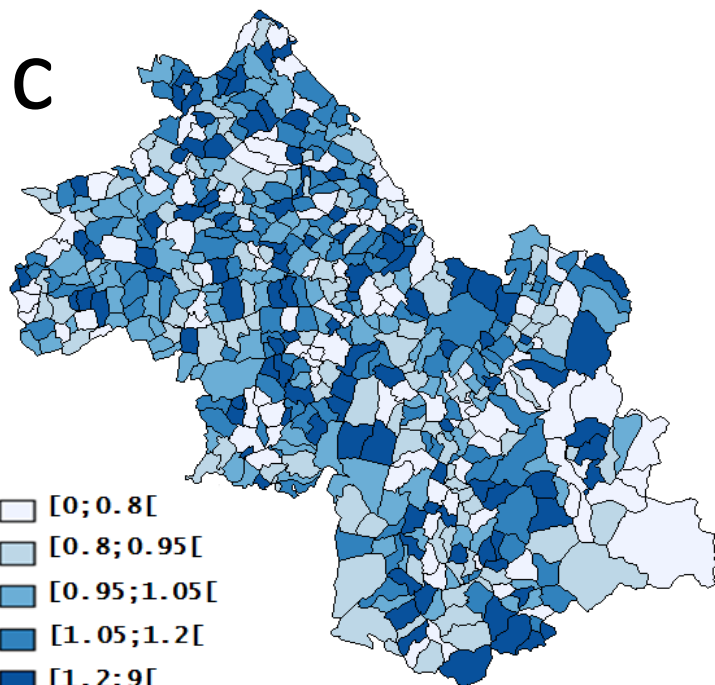**d**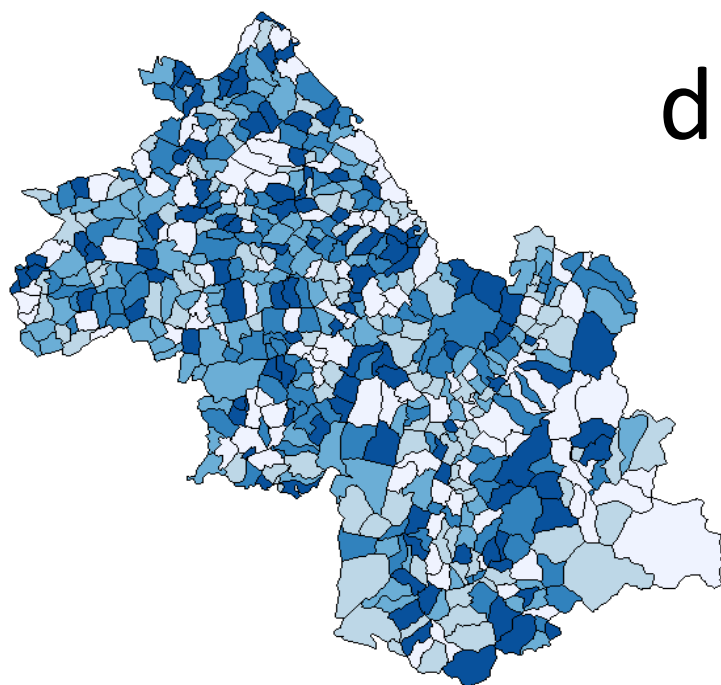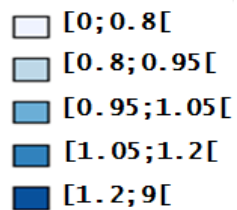

Supplement: Additional file 4: Figure S4. — Clusters of bladder cancer cases found by BYM models: (a) Mapping of the log relative risks estimated by the BYM model using hierarchical Bayesian spatial modeling without adjustment on the Townsend index, (b) Mapping of the log relative risks estimated by the BYM model using hierarchical Bayesian spatial modeling with adjustment on the Townsend index, (c) Mapping of the log relative risks estimated by the M-based BYM model using hierarchical Bayesian spatial modeling with fixed effects, (d) Mapping of the log relative risks estimated by the M-based BYM model using hierarchical Bayesian spatial modeling with random effects. (PDF 214 kb) [file 12874_2016_228_MOESM4_ESM.pdf]

**a**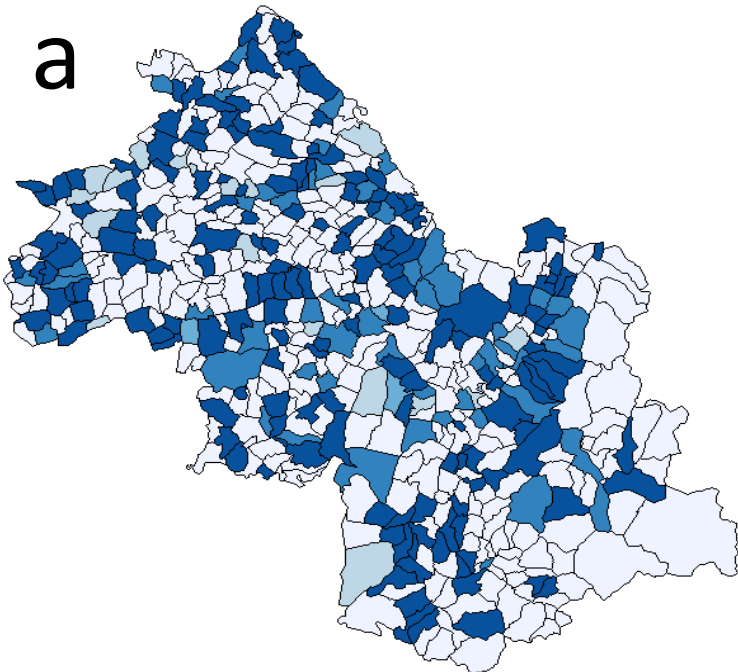**b**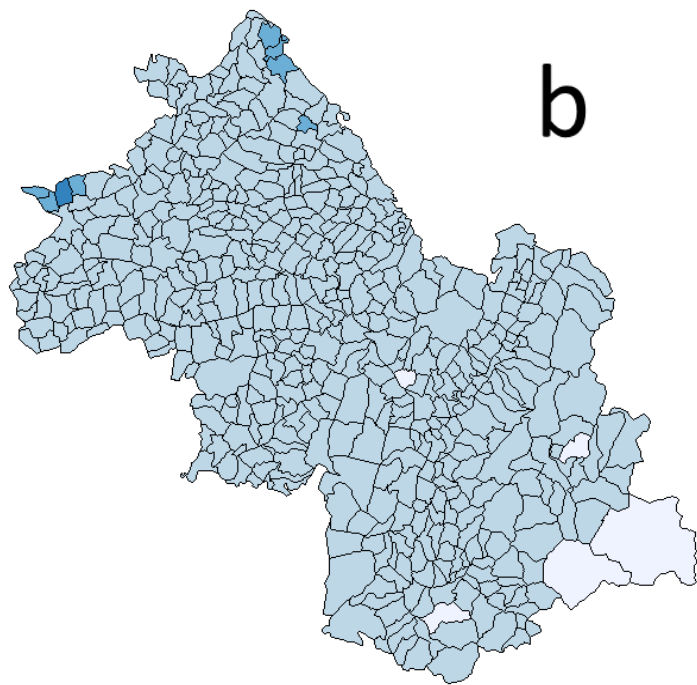**c**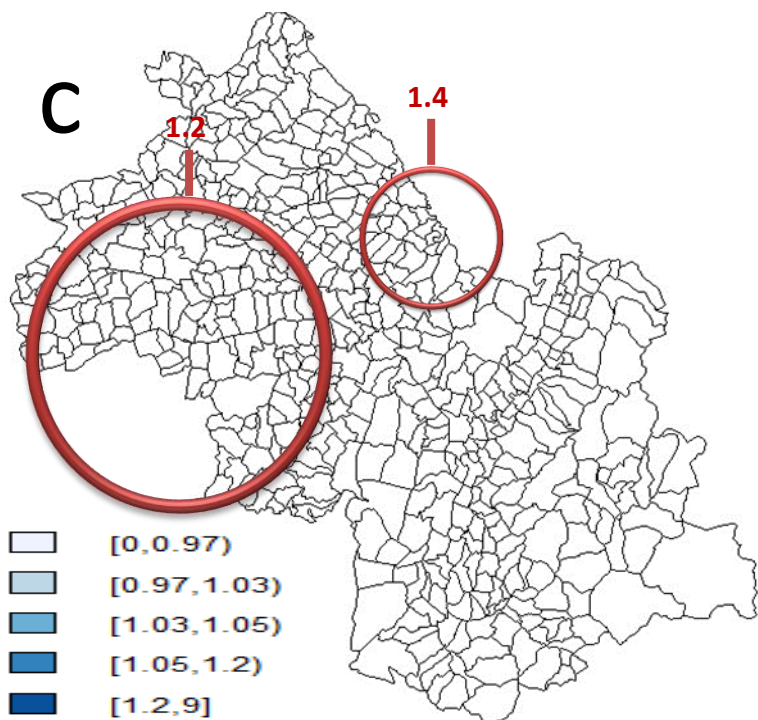**d**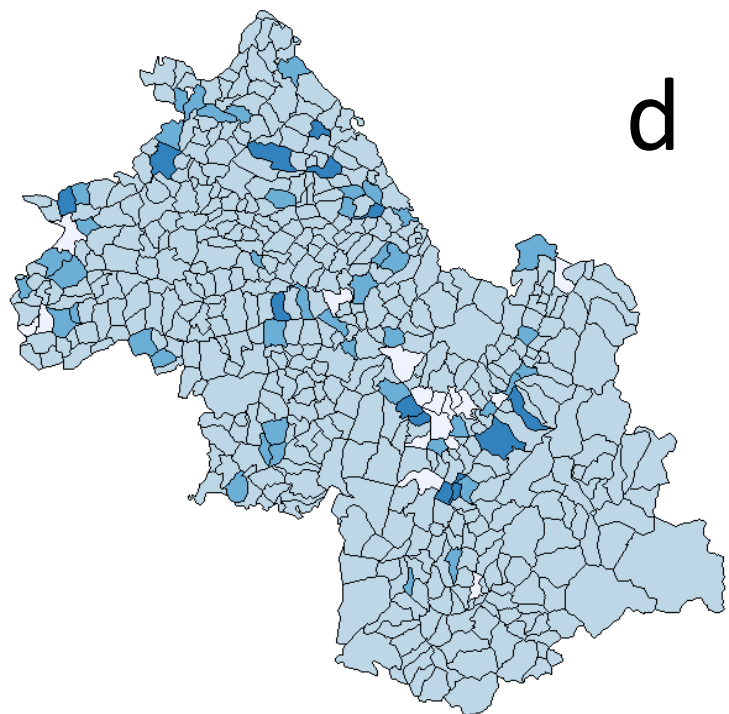

Supplement: Additional file 5: Figure S5. — Clusters of colon-rectum cancer cases found by different methods with alternative cut-points of relative risks: (a) geographic variations of standardized incidence ratio, (b) Mapping of the log relative risks estimated by the CAR model using hierarchical Bayesian spatial modeling without adjustment on the Townsend index, (c) SaTScan clusters without adjustment on the Townsend index (2 clusters of high risk), (d) Mapping of the log relative risks estimated by the heterogeneity model using hierarchical Bayesian spatial modeling without adjustment on the Townsend index (lightest to darkest color). (PDF 367 kb) [file 12874_2016_228_MOESM5_ESM.pdf]
