# Supplementary material for: Impact of socioeconomic inequalities on geographic disparities in cancer incidence: comparison of methods for spatial disease mapping
Source: BMC Med Res Methodol. 2016 Oct 12;16:136. doi: 10.1186/s12874-016-0228-x (PMC5059978; doi:10.1186/s12874-016-0228-x)
Supplement: Additional file 6: Table S1. — Posterior means of the between-disease correlation matrix for the M-based BYM model with random effects. (DOCX 32 kb) [file 12874_2016_228_MOESM6_ESM.docx]

# Additional file 6

Table S1 – Posterior means of the between-disease correlation matrix for the M-based BYM model with random effects

| Cancer | Lung | Prostate | Bladder | Colon-rectum |
| --- | --- | --- | --- | --- |
| Lung | 1 |  |  |  |
| Prostate | 0.00 | 1 |  |  |
| Bladder | 0.42 | 0.16 | 1 |  |
| Colon-rectum | 0.19 | 0.05 | 0.18 | 1 |
